# Effects of hydrophilic extract of *Nasturtium officinale* on prevention of ethylene glycol induced renal stone in male Wistar rats

**DOI:** 10.15171/jnp.2016.23

**Published:** 2016-08-07

**Authors:** Sadrollah Mehrabi, Eslam Askarpour, Farhad Mehrabi, Ramin Jannesar

**Affiliations:** ^1^Medicinal Plant Research center, Yasuj University of Medical Sciences, Yasuj, Iran; ^2^Student Research Committee, Yasuj University of Medical Sciences, Yasuj, Iran; ^3^Student Research Committee, Shiraz University of Medical Sciences, Shiraz, Iran

**Keywords:** *Nasturtium officinale*, Calcium oxalate, Ethylene glycol, Nephrolithiasis

## Abstract

**Background:**

*Nasturtium officinale* is a traditional herb that is used for diuresis.

**Objectives:**

The aim of this study is to determine the effects of hydrophilic extract of *Nasturtium officinale* on ethylene glycol-induced renal stone in male Wistar rats.

**Materials and Methods:**

In this study 32 male Wistar rats were randomly divided in six groups and studied during 30 days. Two groups of negative and healthy control received 1% ethylene glycol in water respectively. Low and high dose preventive groups, in addition to 1% ethylene glycol, daily gavaged with 750 mg/kg and 1.5 g/kg of extract respectively. All rats were hold in metabolic cages individually in days 0, 15 and 30 and 24-hour urine samples were collected and checked for urinary parameters of stone formation. In 30th day, rats were anesthetized with ether, and after taking serum sample from them, were sacrificed and their kidneys were sent for pathological evaluation and for presence and volume of calcium oxalate crystals.

**Results:**

Percentage of calcium oxalate crystals in negative control groups (75%), preventive groups with low dose (28.6%) and high dose (57.1%) in comparison to healthy control group (12.5%) increased (*P* < 0.05). In 30th day urinary oxalate concentration in preventive and negative control groups were more than healthy control group (*P* < 0.05).

**Conclusions:**

This research showed that the *Nasturtium officinale* extract has no significant effects in urinary and chemical parameters efficient in calcium oxalate stone crystals in rat but its extract in low dose has some preventive effect on renal stone formation.

**Implication for health policy/practice/research/medical education:**

In this study 32 male Wistar rats were randomly divided in six groups and studied during 30 days to determine the effects of hydrophilic extract of Nasturtium officinale on ethylene glycol-induced renal stone in male Wistar rats. Results showed that the Nasturtium officinale extract has no significant effects on calcium oxalate stone formation in rats although in low dose has some preventive effect on renal stone formation.

## 1. Background

Prevalence of urinary stones is about 1%-15% (in average 3%-5%) in different populations. The urinary stones are formed most in men, in the third to fifth decade of life and cause acute complications such as flank and abdominal pain, hematuria, and urinary infection (1-3). Xanthogranulomatous pyelonephritis and unilateral and bilateral renal failure could be mentioned among chronic complications (1-4).

Stone former patients are treated by different ways, such as conservative methods (consuming liquids, acid and alkaline materials), surgical methods including relief of obstruction, percutaneous nephrolithotomy, transurethral lithotripsy and open surgery (1,3,5).

In spite of significant progress in diagnosis and treatment of urinary stones, the rate of recurrence is high (about 50% during 5 years) that is due to inefficacy of current methods in changing pathophysiology and course of stone formation. Therefore by providing interventional methods, an important step can be taken in prevention and prolonged treatment of urinary stones (6-10).

Nowadays due to side effects of chemical drugs, using herbal medicines has attracted the attention of contemporary researchers (11,12).

The plants *Nasturtium officinale* belong to Liliace family and grow on the 1800-2600 meters altitudes of Zagros mountains. Native people of this region use the aerial parts of the plant for the treatment of abdominal pain, rheumatic pain and urinary stones. Also in recent studies the analgesic effect *Nasturtium officinale* has been shown (4,5). Furthermore, there are some steroids in its bulb, which have shown cytotoxic and cytostatic effects against malignant tumor cells (4).

## 2. Objectives

In spite of long-lasted administration of this plant by native people as a treatment for renal stones, no scientific research has been conducted on its effects on renal stones. Therefore we aimed to study the effects of aerial parts of the plant extract on preventing calcium oxalate renal stones induced by ethylene glycol in male Wistar rats.

## 3. Materials and Methods

This experimental study was performed in 2013 in Medicinal Plant Research Center, Yasuj University of Medical Sciences. Thirty-two weanling male Wistar rats with a weight range of 120-180 g were randomly divided into four groups of eight rats and studied during 30 days.

Group1, received daily distilled water without receiving ethylene glycol (healthy control), and group two received distilled water in addition to ethylene glycol

1% (v/v) in drinking water (negative control). In addition to 1% ethylene glycol in drinking water, groups 3 and 4 received 750 mg/kg and 1.5 g/kg daily *Nasturtium officinale* extract respectively.

Rats were acclimatized in stainless steel metabolic cages for one week, and maintained under the temperature of 25±2°C and 12-hours dark-light cycle and 50% humidity during the study period.

One percent ethylene glycol was used to induce calcium oxalate renal stones. The stone forming dosage of ethylene glycol was determined after observing that 1% ethylene glycol in drinking water induced calcium oxalate crystals in 80% of rat’s kidneys.

### 3.1. Measurements

#### 3.1.1. Urine analysis

24-hour urine samples of all rats were collected in metabolic cages individually in days 1, 15 and 30 of the study and urine volume, oxalate, citrate, calcium, phosphorous and uric acid were determined in laboratory.

#### 3.1.2. Hematologic and pathologic variables

After 30thday, rats were anesthetized with ether and after taking blood samples from their heart for measuring calcium, uric acid and creatinine, they were sacrificed and their kidneys removed. Right kidneys were weighed and for defining the interstitial tissue water incubated in 80˚C for 24 hours and weighed again.

#### 3.1.3. Calcium oxalate deposits

Left kidneys were fixed within 10% formalin and cut into 5 µm sections in pathology laboratory using CUTIX microtome (Klinpath, Netherland).

Hematoxylin and eosin were used for tissue staining. Then in each section, 10 microscopic fields with magnification of 40×10 were randomly selected in equal numbers in cortex and medulla, and numbers of calcium oxalate crystals (number of renal tubes containing these crystals) were counted.

### 3.2. Nasturtium officinale extract

The plant - *Nasturtium officinale* – was collected in spring from the Yasuj mountains and coded in herbarium by Herbarium number 3225. The aerial part of plant was washed in cold water, dried in shade and room temperature and then grinded using Lacimbali grinder (Italy). Then 500 g of grinded dried plant was soaked in distilled water and filtered after 48 hours. The filtered liquid was concentrated by rotary at 50°C and under vacuum condition. The resulted concentrate was incubated in 50°C to be completely dried. Two hundred five grams of dried extract were obtained from 500 g of dry plant. The obtained extract was kept in refrigerator and desired concentrations were prepared in distilled water to be gavaged to rats.

### 3.3. Ethical issues

The research followed the tenets of the Declaration of Helsinki. The research was approved by ethical committee of Yasuj University of Medical Sciences. Prior to the experiment, the protocols were confirmed to be in accordance with the Guidelines of Animal Ethics Committee of Yasuj University of Medical Sciences.

### 3.4. Statistical analysis

Statistical calculation: the data were analyzed by SPSS software version 15 and groups’ means were compared using one-way analysis of variance (ANOVA) with Scheffe post hoc test *to compare groups in pairs***.**

## 4. Results

### 4.1. Measurement of urinary variables

Urinary oxalate levels are shown in Table 1, for the first, 15th and 13th days of the study. The mean levels of urinary oxalate are increased at 15th and 30th days in all groups receiving ethylene glycol. The differences were not statistically significant (*P*>0.05).

**Table 1:** Mean and standard deviation of urine oxalate levels (nmol/24 h) at first, 15th and 30th days in studied groups

**Group**	**Number**	**Time**		
		**Day 1**	**Day 15**	**Day 30**
Healthy control	8	0.54 ± 0.09	0.60 ± 0.12	0.58 ± 0.10
Negative control	8	0.51 ± 0.21	0.59 ± 0.13	0.67 ± 0.21
Low dose	8	0.56 ± 0.15	0.57 ± 0.17	0.64 ± 0.15
High dose	8	0.56 ± 0.12	0.69 ± 0.07	0.61 ± 0.15
P value		0.924	0.354	0.375

In the day of 15th, the lowest rate of urinary oxalate was seen in group receiving low dose and the highest level was seen in group receiving high dose that has statistically significant difference (*P*<0.05).

In the third measurement (day 30th) the level of urinary oxalate in the prevention groups with low dose (0.64±0.15 nmol/24 h) and high dose (0.61±0.15 nmol/24 h) and negative control group (0.67±0.21 nmol/24 h) were more in comparison to healthy control group (0.58±0.10 nmol/24 h), but there was no significant difference between them (*P*>0.05).

Study of other measured urinary parameters such as 24-hour volume, citrate, pH, calcium, phosphorous, creatinine and uric acid did not show statistically significant difference between the groups.

### 4.2. Serum biochemical parameters

The level of serum parameters was shown in Table 2. The lowest level of calcium, phosphorus, uric acid and creatinine was observed in prevention group with high dose in comparison to other groups but there was not difference statistically significant (*P*>0.05).

**Table 2:** Mean and standard deviation of serum biochemical parameters (mg/dL for all parameters) in studied groups

**Group**	**Variable**			
	**Calcium**	**Phosphorus**	**Uric acid**	**Creatinine**
Healthy control	9.70 ± 1.44	0.47 ± 8.47	0.56 ± 2.35	0.03 ± 0.57
Negative control	0.80 ± 9.76	0.72 ± 8.38	0.41 ± 2.17	0.03 ± 0.57
Low dose	0.38 ± 9.45	0.59 ± 8.38	0.48 ± 2.16	0.05 ± 0.57
High dose	0.18 ± 9.14	0.59 ± 7.84	0.70 ± 2.12	0.02 ± 0.56
F	0.658	1.409	0.268	0.126
*P* value	0.586	0.264	0.848	0.944

### 4.3. Levels of calcium oxalate crystal depositions in kidney

The highest number of crystal depositions were observed in negative control group (85%) which is statistically significant difference with other groups including healthy control (12.5%), low dose (28.6%) and high dose (42.9%) prevention groups (*P*<0.05). The lowest number of crystal depositions were in low dose prevention group (28.6%).

## 5. Discussion

Although great development has occurred in treatment of urinary tract stones by advancement of technology, but still there is no effective and harmless pharmaceutical method which leads to perfect treatment or prevention of urinary stone formation (1,3,12,14).

Different herbal and industrial medicines have been marketed to reduce stone formation, which may be relatively effective on prevention and treatment of calcium and uric acid stones but some controversy are exist about their efficacy in different studies (4,15,16). While, many people of south part of Iran believe that *Nasturtium officinale* has efficacy for treatment of urinary stones and use it, the aim of this study was to study the effect of *Nasturtium officinale* extract on the calcium oxalate stones induced by ethylene glycol in male Wistar rats.

The mean levels of urinary oxalate are increased at 15th and 30th days in all groups receiving ethylene glycol and also in the healthy control. Urinary parameters such as 24-hour volume, citrate, pH, calcium, phosphorous, creatinine and uric acid did not show statistically significant difference between the groups. Lowest level of calcium was in prevention group with high dose and highest level of calcium was in negative control group. The highest number of crystal depositions was observed in negative control group in comparison to other groups.

In a study conducted by Atmani et al in 2007, the effect of *Cynodon dactylon* on renal stone was studied. In this study ethylene glycol was used as stone inducing substance. Except urinary oxalate in prevention group, and calcium and sodium in treatment group, the extract had no effect on other urinary parameters, but caused a reduction in level of the crystal depositions in kidney, which shows its preventive effect on renal stone formation (14).

Also in a study conducted by Hajzadeh et al in 2006, the effect of alcoholic extract of *Nigella sativa* on ethylene glycol induced renal stone in rat model was studied. The findings of this research showed that the alcoholic extract of *Nigella* has preventive effect on calcium oxalate crystal depositions in kidney (10).

Our study is in agreement with previous studies, and in the extract receiving groups, decrease in calcium oxalate crystal depositions in kidney is observed especially in preventive group with low dose extract. The decreased number of crystals was not dose dependent and in prevention group with high dose of extract, more crystals were observed.

In a study by Akanae et al, the protective effect of *Orthosiphon grandiflorum* on calcium oxalate stone formation was studied. They found that *Orthosiphon grandiflorum* has a significant inhibitory effect on crystal depositions in the calcium oxalate stone forming rat model (15).

Also in a study by Moriyama et al, the inhibitory effect of *Quercus salicina* extract on urinary oxidative stress and level of urinary calcium in rat ca-ox renal stone model was studied. The result of this study showed that the extract of *Quercus salicina* has antioxidative effect and could prevent renal stone formation (16).

The results of these two studies were not in consistency with the result of our study, because in contrast with these studies, in our study the extract had no effect on decrement of calcium crystal deposition and reducing the level of stone formation.

Also according to the results of our study, the extract of *Nasturtium officinale* had no significant effect on pH, Ca, creatinine, uric acid and 24-hour volume.

Although there is not any study regarding efficacy of *Nasturtium officinale* extract in treatment and prevention of renal stones, but in contrast to above-mentioned studies that show effect of *Quercus salicina* and *Cynodon dactylon* on urinary stones and in spite of domestic believe about effectiveness of *Nasturtium officinale* on renal stones its extract has no effect on the most urinary factors effective in formation of urinary stones such as urine volume, and urinary calcium, phosphor and uric acids that are the most important metabolic factors efficient in formation of urinary stones. Also it increases urinary concentration of oxalate that may be due to synergistic effect of ethylene glycol with extract of *Nasturtium officinale*.

Additionally, in a research by Khaksarian et al, the analgesic effect of *Allium jesdianum* extract was studied. This research showed that *Allium jesdianum* consists of morphine in addition to quinolone and benzene and has analgesic effect (13).

In our research the analgesic effect of *Nasturtium officinale* was not studied, but the result of Khaksarian et al research may support its efficacy on relieving pains of urinary stone passage.

In a study by Vahdani et al preventive and therapeutic effects of *Allium jesdianum* extract was studied. Their results showed that *Allium jesdianum* extract has no preventive effect in calcium oxalate stone in Wistar rat and increased deposition of calcium oxalate crystals in kidney (20). Results of this study is somewhat consistent with our results although watercress decreased deposition of calcium oxalate crystals in kidney in low and high dose extract.

Shafaeipour et al studied effects of *Alhagi maurorum* on prevention and treatment of ethylene glycol induced renal stone in male Wistar rats. They found that this extract by decreasing urinary oxalate concentration and increasing urinary citrate level are efficient in prevention of calcium oxalate crystals in kidney (21). Our results are not consistent with this study because the extract of *Nasturtium officinale* had no effect on urinary parameters and stone formation.

## 6. Conclusions

The results of this research showed that the *Nasturtium officinale* extract has no significant effects in urinary and chemical parameters efficient in calcium oxalate stone formation in rat but its extract especially in low dose has some preventive effect on renal stone formation and crystal deposition that need more studies.

## Authors’ contribution

AA and AAO conducted the research. AA, AAO and LK analyzed the data and prepared the pri­mary draft. AA, AAO, LK, RS and SM conducted the experimental measurements.

## Conflicts of interests

The authors declared no competing interests.

## Funding/Support

This study is labeled as Student Research Project # 93p88, and was supported financially by the Student Research Committee of Yasuj University of Medical Sciences, Yasuj, Iran.
